# Supplementary material for: Highdicom: a Python Library for Standardized Encoding of Image Annotations and Machine Learning Model Outputs in Pathology and Radiology
Source: J Digit Imaging. 2022 Aug 22;35(6):1719–37. doi: 10.1007/s10278-022-00683-y (PMC9712874; doi:10.1007/s10278-022-00683-y)
Supplement: Supplementary file 1 — Supplementary file1 (PDF 5.84 MB) [file 10278_2022_683_MOESM1_ESM.pdf]

Supplementary material for “Highdicom: A Python library for standardized encoding of image annotations and machine learning model outputs in pathology and radiology”

April 23, 2022

Experimental image datasets

Preparation of slide microscopy images and annotations in DICOM format

We downloaded SM images in SVS format from either the Genomics Data Commons (GDC) Data Portal using the GDC Data Transfer Tool software or from TCIA via the CPTAC histopathology interface in case of TCGA and CPTAC collections, respectively. In addition, we downloaded the corresponding biospecimen and clinical metadata in JSON format via the GDC Data Portal for both TCGA and CPTAC collections and additional metadata in JSON format via the CPTAC Clinical Data API for the CPTAC collections. For each image contained in a given SVS file, we created a DICOM VL Whole Slide Microscopy Image instance and stored it in a DICOM Part 10 file. Briefly, we copied image pixel data contained in TIFF tiles as well as relevant pixel-related metadata contained in TIFF tags (e.g., `ImageLength`, `ImageWidth`, `ImageDescription`, `Compression`, and `PhotometricInterpretation`) into the corresponding DICOM data elements. We further enriched DICOM data sets with information extracted from TCIA biospecimen metadata JSON documents, e.g. fixatives and embedding media used for specimen preparation as well as relevant patient, study, and specimen identifiers. For identifiers, we followed the same patterns used for the radiology data sets to facilitate matching of pathology and radiology DICOM data sets.

As described in the Methods section, we structured the content of the SR documents based on template TID 1500 “Measurement Report” and encoded image annotations using content items defined either in template TID 1410 “Planar ROI Measurements and Qualitative Evaluations” in case of graphical ROI annotations or TID 1501 “Measurement and Qualitative Evaluation Group” in case the annotations applied to an entire image or series. In either case, we included content item “Finding” (DCM 121071) to encode the concept that the given image or image region represents, e.g., “Neoplasm” (SCT 108369006). We further extended template TID 1501 and included additional content items to encode measurements and qualitative evaluations of the image or image region that are specific to cancer diagnosis in pathology or radiology. We included content items “Morphology” (SCT 116676008) and “Topography” (SCT 116677004) with value type `CODE` to encode the tumor histomorphology (squamous cell carcinoma, adenocarcinoma, etc.) and tissue of origin (bronchus, upper lobe, etc.) using the International Classification of Diseases for Oncology (ICD-O-3) and the Clinical Modification of the International Classification of Diseases (ICD-10-CM) coding systems, respectively. In addition, we included content items “Percent tumor cells” (caDSR 5432686), “Percent tumor nuclei” (caDSR 5455534), and “Specimen necrosis” (caDSR 5455511) with value type `NUM` to provide measurements of the percentage of viable or dead tumor tissue. We encoded graphical annotations of image regions of interest (ROIs) as vector graphics in

DICOM Comprehensive SR or Comprehensive 3D SR documents as described above. For SM images used in the pathology experiments, graphical annotations of tumor regions were not available in TCIA, and we therefore encoded image-level annotations in DICOM Comprehensive SR documents using TID 1501 “Measurement and Qualitative Evaluation Group”. However, we annotated a subset of SM images using the Slim viewer ¹ and encoded the resulting ROIs as content items with type SC00RD3D in DICOM Comprehensive 3D SR documents using TID 1410 “Planar ROI Measurements and Qualitative Evaluations”.

Preparation of computed tomography images and annotations in DICOM format

For the radiology experiments, we used the LIDC-IDRI dataset of 1018 diagnostic and screening CT studies from multiple institutions. We downloaded CT images, consisting of a single axial series per study, in DICOM format from TCIA using the NBIA Data Retriever software. Each scan in the LIDC-IDRI dataset is annotated in the form of segmentation masks for lung nodules. Each scan contains one or more annotated nodules, and each nodule may be annotated by multiple readers. The annotations for this dataset were already available in the original custom XML-based format, and also as DICOM Segmentation images and DICOM SR documents that were created in prior work using the C++-based DCMQI toolkit [1, 2]. For the purpose of demonstrating a full workflow within the Python programming language, we re-created annotations in both DICOM Segmentation and SR format from the original XML annotations, largely following Fedorov et al. [1, 2], using *highdicom* and used the resulting datasets (rather than those created with DCMQI) for the subsequent experiments. The SEG images are hereby used to encode the annotation of a single nodule by a single reader as a single segment, with a Segmented Property Category of “Morphologically Abnormal Structure” (SCT 49755003) and Segmented Property Type “Nodule” (SCT 27925004). The SR documents used the Comprehensive3DSR IOD and included measurements and qualitative evaluations of each of the regions in the SEG images, referenced via a content item of type IMAGE. Measurements of each nodule ROI included the “Volume” (SCT 118565006) and “Diameter” (SCT 81827009) as NUM content items, and qualitative evaluations included the subtlety, internal structure, calcification, sphericity, margin, lobulation, spiculation, texture, and malignancy of the nodule using a range of coded concepts as suggested by [1, 2]. Note that these measurements and qualitative evaluations were not used during model training, but were included in the annotation SR for the sake of completeness. The script used for conversion is publicly available².

Validation of DICOM data sets

We evaluated the compliance of prepared data sets with the DICOM standard using both automated validation tools and manual expert review. Specifically, we used the `dciodvfy` command line tool of the *dicom3tools* ³ package to assert that individual DICOM files are structured according the corresponding Information Object Definition (IOD) and the `DicomSRValidator` program of the *PixelMed Java DICOM Toolkit* ⁴. We further used the `dcdump` and `dcsrdump` command line tools of the *dicom3tools* package and the `dcmdump` command line tool of the *DICOM toolkit (DCMTK)* ⁵ package to manually review and validate the content of DICOM files.

¹<https://github.com/mghcomputationalpathology/slim>

²https://url_redacted_for_review

³<http://www.dclunie.com/dicom3tools.html>

⁴<http://www.pixelmed.com/dicomtoolkit.html>

⁵<https://dcmtk.org/>

Further model training details

We implemented the deep convolutional neural network models using the *PyTorch* Python library [3] and trained them on a Linux supercomputer with NVIDIA V-100 graphical processing units (GPUs) using the *CUDA* and *cuDNN* C++ libraries. We optimized model parameters using Adam optimizer with momentum and optimized the learning rate as well as other hyperparameters using random search.

The data preprocessing pipelines were implemented in form of classes derived from `torch.data.Dataset`, which load the images and corresponding image annotations from DICOM files into *NumPy* arrays, transform the data in memory into the representation expected by the respective model, and return a pair of image frames and labels as *PyTorch* tensors. Instances of these classes are instantiated given the location of DICOM files on disk as well as the SOP Instance UIDs of DICOM SR documents, which contain annotations that are considered the ground truth for model training as well as references to the source images from which the annotations were derived. For each example, the `__getitem__` method loads image frames and annotations from DICOM files on disk into memory as `numpy.ndarray` objects, transforms the data into the representation expected by the first layer of the neural network (optionally performing data augmentation), and returns the pair of transformed pixel data and associated labels as a tuple of `torch.tensor` objects.

Model evaluation

We selected one pathology and radiology model on a validation set and evaluated the performance of selected models on a hold-out test set.

For pathology, we developed classifiers to categorize whole slide images into either normal lung tissue, lung adenocarcinoma, or lung squamous cell carcinoma. To this end, we thresholded the probabilistic fractional segmentation images outputted by the neural network model (using a threshold value of 0.5 probability) for each class and used thereby generated binary segmentation masks to compute the relative tumor area, i.e., the ratio of the number of image frames classified as tumor and the number of image frames classified as tissue. Based on these aggregated measurements, we created binary whole slide image classifiers to distinguish normal lung from non-small cell lung cancer (NSCLC, lung adenocarcinoma or lung squamous cell carcinoma), lung adenocarcinoma (LUAD), or lung squamous cell carcinoma (LUSC). The optional threshold for each classifier was determined via Receiver Operator Characteristic (ROC) analysis (Supplementary Figure S1A). Evaluating the classifiers at the selected threshold values, we achieved an accuracy of 0.98 for separating normal lung from NSCLC (Supplementary Figure S1B), a high correlation between the predicted relative tumor area and the annotated tumor cell percentage for NSCLC examples (Supplementary Figure S1C), and an accuracy of 0.85 for distinguishing between LUAD and LUSC (Supplementary Figure S1D).

For radiology, we evaluated the CT lung nodule detection model in terms of study-level results. Nodule sensitivity (recall) was calculated as the fraction of annotated nodules for which any predicted box overlapped any reader’s annotated with intersection-over-union (IoU) of 0.5 or greater on any frame. False positives in neighboring frames were clustered together if their IoU (in the x and y directions only) was greater than 0.5, and assigned the score of the highest score in the cluster. This gave an average precision metric of 0.582 for nodule detection. The free-response receiver operating characteristic (FROC), which plots nodule sensitivity (recall) against the average number of false positives per study as the detection score threshold is varied (Supplementary Figure S2).

Visualization of Outputs

Figures S3 and S4 contain examples of image annotations and model outputs visualized within open source tools.

References

- [1] A. Fedorov, M. Hancock, D. Clunie, M. Brockhhausen, J. Bona, J. Kirby, J. Freymann, H. Aerts, R. Kikinis, and F. Prior. *Standardized representation of the TCIA LIDC-IDRI annotations using DICOM*. Tech. rep. The Cancer Imaging Archive, 2018.
- [2] A. Fedorov, M. Hancock, D. Clunie, M. Brochhausen, J. Bona, J. Kirby, J. Freymann, S. Pieper, H. J. W. L. Aerts, R. Kikinis, and F. Prior. “DICOM re-encoding of volumetrically annotated Lung Imaging Database Consortium (LIDC) nodules”. In: *Medical Physics* 47.11 (2020), pp. 5953–5965.
- [3] A. Paszke, S. Gross, F. Massa, A. Lerer, J. Bradbury, G. Chanan, T. Killeen, Z. Lin, N. Gimsheine, L. Antiga, A. Desmaison, A. Kopf, E. Yang, Z. DeVito, M. Raison, A. Tejani, S. Chilamkurthy, B. Steiner, J. Fang L. Bai, and S. Chintala. “PyTorch: An Imperative Style, High-Performance Deep Learning Library”. In: *Advances in Neural Information Processing Systems* 32. Curran Associates, Inc., 2019, pp. 8026–8037.
- [4] A. Fedorov, R. Beichel, J. Kalpathy-Cramer, J. Finet, J.-C. Fillion-Robin, S. Pujol, C. Bauer, D. Jennings, F. Fennessy, M. Sonka, J. Buatti, S. Aylward, J. V. Miller, S. Pieper, and R. Kikinis. “3D Slicer as an image computing platform for the Quantitative Imaging Network”. In: *Magnetic Resonance Imaging* 30.9 (2012), pp. 1323–1341.

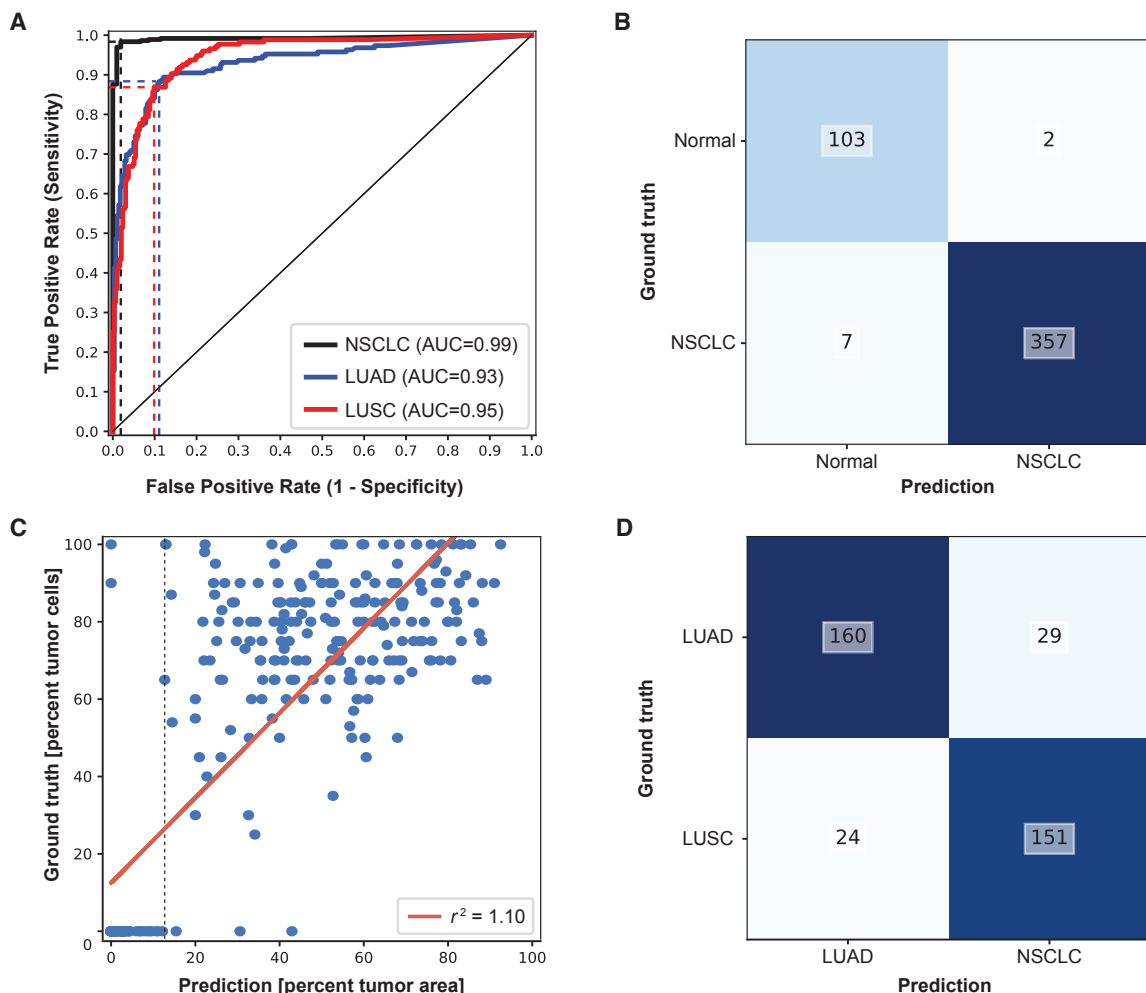

Figure S1: Performance of SM image classifiers (NSCLC — non-small cell lung cancer, LUAD — lung adenocarcinoma, LUSC — lung squamous cell carcinoma). **A** Receiver operating characteristic curve for each binary classification problem. **B** Confusion matrix comparing the ground truth against predicted class labels for the classification of normal lung versus non-small cell lung cancer using the threshold selected in A. **C** Correlation between annotated percent tumor cells and predicted percent tumor area considering examples that were classified as non-small cell lung cancer. **D** Confusion matrix comparing the ground truth against predicted class labels for the classification of lung adenocarcinoma versus lung squamous cell carcinoma considering examples that were correctly classified as non-small cell lung cancer.

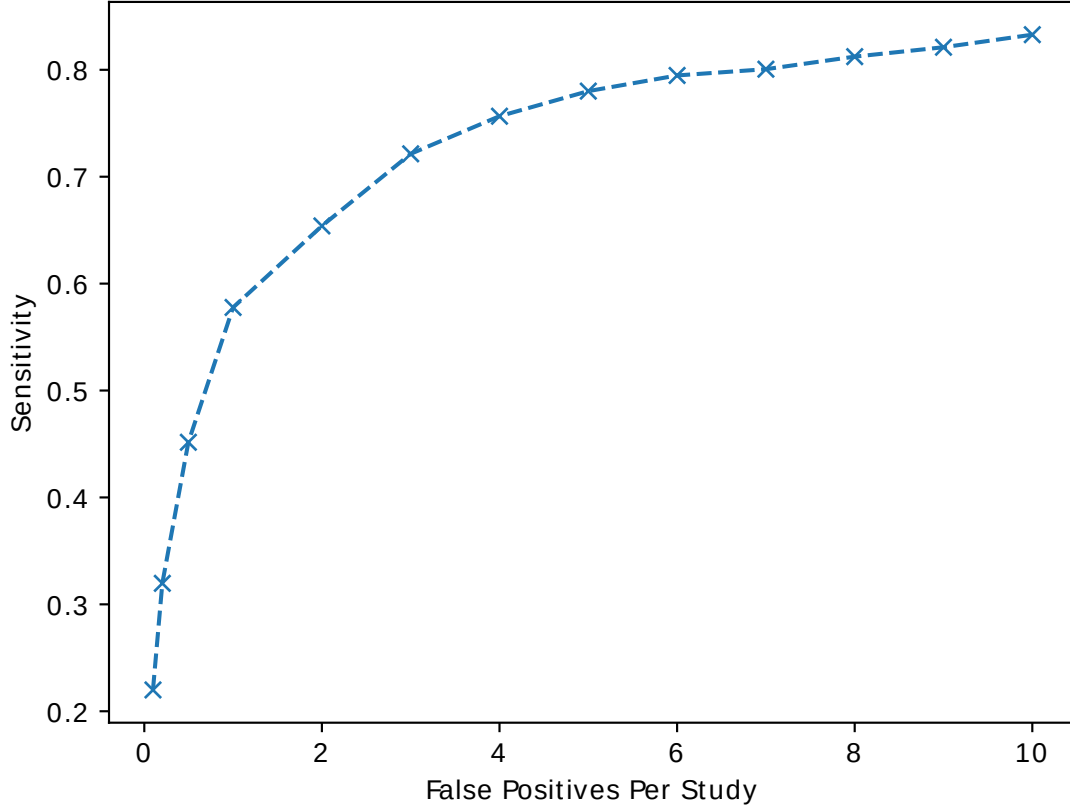

Figure S2: Free-response receiver operating characteristic for the CT lung nodule detection model.

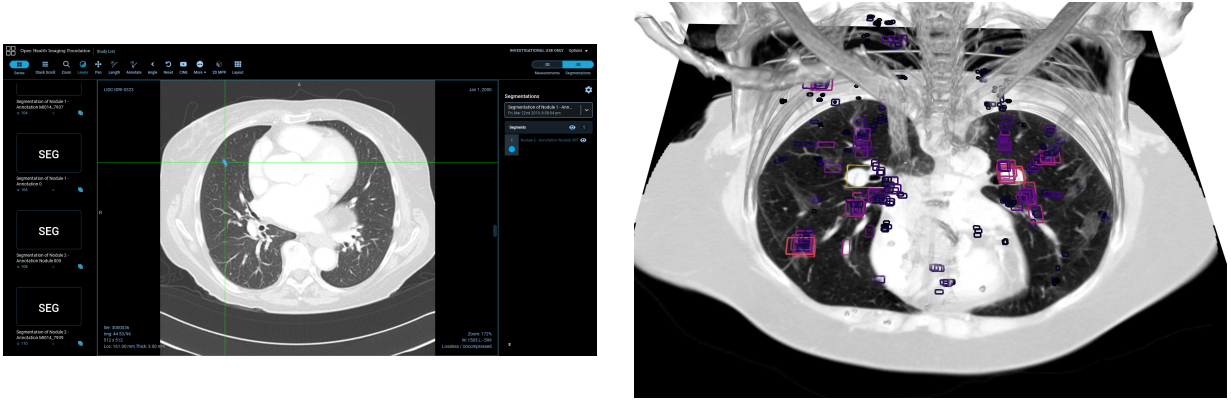

Figure S3: **A** Lung nodule annotation in a CT image of the LIDC-IDRI collection encoded as a segment in a DICOM Segmentation image using highdicom and displayed in the open-source OHIF viewer. **B** Bounding box detection output from the CT lung nodule detection model encoded as a DICOM SR document and visualized using the open-source 3D Slicer software and the Quantitative Reporting extension [4]. Detected bounding boxes are shaded by their detection scores from purple (very low score) to yellow (high score).

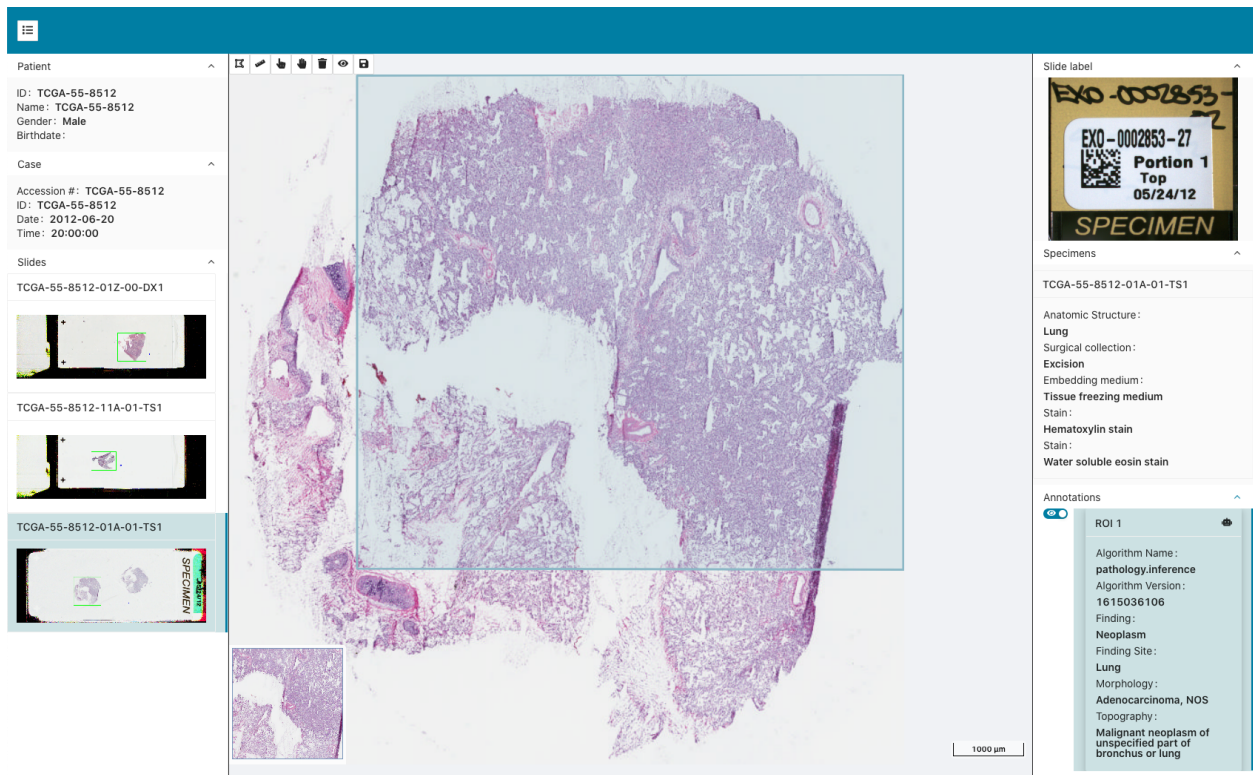

Figure S4: Lung adenocarcinoma regions detected in a slide microscopy image of the TCGA-LUAD collection encoded as SCOORD3D content items in a DICOM Comprehensive 3D SR document using *highdicom* and displayed in the open-source SlIM viewer.
